# *CLCN1* Molecular Characterization in 19 South-Italian Patients With Dominant and Recessive Type of Myotonia Congenita

**DOI:** 10.3389/fneur.2020.00063

**Published:** 2020-02-06

**Authors:** Chiara Orsini, Roberta Petillo, Paola D'Ambrosio, Manuela Ergoli, Esther Picillo, Marianna Scutifero, Luigia Passamano, Alessandro De Luca, Luisa Politano

**Affiliations:** Cardiomiology and Medical Genetics, Department of Experimental Medicine, University of Campania “Luigi Vanvitelli”, Naples, Italy

**Keywords:** *CLCN1* mutations, myotonia congenita, Becker myotonia, Thomsen myotonia, southern Italy

## Abstract

Myotonia congenita is a genetic disease characterized by impaired muscle relaxation after forceful contraction (myotonia). It is caused by mutations in the *CLCN1* gene, encoding the voltage-gated chloride channel of skeletal muscle, ClC-1. According to the pattern of inheritance, two distinct clinical forms have been described, Thomsen disease, inherited as an autosomal dominant trait and Becker disease inherited as an autosomal recessive trait. We report genetic and clinical data concerning 19 patients−13 familial and six isolated cases—all but one originating from the Campania Region, in southern Italy. Twelve patients (63.2%) present Becker type myotonia and 7 (36.8%) Thomsen type. Sex ratio M:F in Becker type is 6:6, while in Thomsen myotonia 4:3. The age of onset of the disease ranged from 2 to 15 years in Becker patients, and from 4 to 20 years in Thomsen. Overall 18 mutations were identified, 10 located in the coding part of the gene (exons 1, 3, 4, 5, 7, 8, 13, 15, 21, 22), and four in the intron part (introns 1, 2, 10, 18). All the exon mutations but two were missense mutations. Some of them, such as *c.2551 G* > *A, c.817G* > *A* and *c.86A* > *C* recurred more frequently. About 70% of mutations was inherited with an autosomal recessive pattern, two (c.86A and *c.817G*>*A*) with both mechanisms. Three novel mutations were identified, never described in the literature: p.Gly276Ser, p.Phe486Ser, and p.Gln812^*^, associated with Becker phenotype. Furthermore, we identified three *CLCN1* mutations—c.86A>C + c.2551G > A, c.313C > T + c.501C > G and 899G > A + c.2284+5C > T, two of them inherited *in cis* on the same allele, in three unrelated families. The concomitant occurrence of both clinical pictures—Thomsen and Becker—was observed in one family. Intra-familial phenotypic variability was observed in two families, one with Becker phenotype, and one with Thomsen disease. In the latter an incomplete penetrance was hypothesized.

## Introduction

Myotonia congenita is a genetic disease characterized by impaired muscle relaxation after forceful contraction (myotonia). The term “myotonia” indicates the main characteristic of this pathology, i.e., the presence of the so-called *myotonic phenomenon*, usually defined as “the delay in muscle relaxation after a prolonged contraction” (1, 2). The diseases characterized by the presence of the “myotonia” can be subdivided into two large groups (3): Myotonic Dystrophies (DM), characterized by the *presence* of progressive muscular atrophy and weakness, and multi-systemic involvement and Non-dystrophic myotonias (NDM), characterized by the *absence* of progressive muscle atrophy and weakness, and multi-systemic involvement. The latter include the so-called *Muscular Channelopathies*, due to mutations in genes that code for proteins in the channels of sodium, chlorine, calcium, and potassium, some of which may be responsible for both myotonic and periodic paralysis.

Myotonia congenita is the most common muscle channelopathy. First described by Thomsen in 1876 as an autosomal dominant disease (4), a monograph on the pathology was published in 1907 by Sergio Pansini, a medical doctor at the University of Naples. The recessive form was described by PE Becker in 1963 (5). Their prevalence is estimated in 1: 100,000 live births, with onset in both childhood and adulthood (2). Depending on whether the mutation is present on both alleles or only on one of them, the clinical pictures of Becker's myotonia (BM, AR), or Thomsen myotonia (TM, AD), are observed. Both diseases are characterized by muscle stiffness, warm-up or heating (movement improves with repetition), trigger events (cold, stress, and exercise). Pregnancy and menstruation can worsen the symptoms. Usually patients with Becker's myotonia present a more severe clinical picture (1).

Myotonia congenita is due to mutations in the *CLCN1* gene (ClC-1 chlorine channel), located on the long arm of chromosome 7, in position 7q34. *CLCN1* (OMIM # 118425) consists of 23 exons and has a transcript of 3,093 nucleotides. ClC-1 protein, consisting of 988 amino acids, represents the main voltage-dependent chloride channel in skeletal muscle cells and stabilizes the resting potential of the membrane (6). When the Cl^−^ ion conductance falls below 40%, an accumulation of K^+^ ions in the T tubules occurs, with consequent depolarization of the cell membrane and appearance of myotonia (7). More than 275 variants, causative of myotonia congenita, have been identified; about 95% of them are point mutations, while 1–5% deletions or duplications (8). Most of these mutations cause Becker myotonia, while only about 20 pathogenetic variants have so far been associated with Thomsen myotonia. At least 12 mutations can cause both pathologies for different pathogenetic mechanisms, as variants with dominant-negative effect, reduced penetrance, incomplete dominance, aplotypic background, differences in helix expression or, more simply, inability of current techniques to identify the second pathogenic variant (2). In a simplified way, mutations located at the fast gates level are recessive phenotype mutations, whereas mutations located at the slow gates level are dominant phenotype mutations (9). Recessive mutations may affect the structure of the ClC-1 protein, alter its transport, or disable the formation of dimers (10, 11); on the contrary dominant mutations have a dominant negative effect on dimerization (10). The diagnosis is based on clinical data, serum creatine kinase (CK) levels, usually normal or only slightly increased between 3 and 4 times the upper reference limit, presence of myotonic discharges on the EMG, and genetic analysis.

The current therapy is based on the use of drugs such as Mexiletine (12), Phenytoin, Carbamazepine and Acetazolamide (13, 14). However, studies in progress are evaluating new-generation molecules (13, 15), and “gating” or “traffic” corrector drugs through the so-called *pharmacological chaperones* (16, 17). We report genetic and clinical data concerning 19 patients−13 familial and 6 isolated cases—all but one originating from the Campania Region, in southern Italy.

## Methods

### Clinical Diagnosis

We investigated 19 patients−13 of them from 7 families—with clinically defined Myotonia congenita. All patients were referred to our clinics due to variable increases of creatine kinase (CK) or grades of muscle stiffness. At the time of the first examination, information about family history, age of onset of symptoms, current therapy was collected. All patients underwent skeletal muscle and cardiological evaluation, spirometry, routine hematochemistry, and muscle enzymes. EMG was available in 6/19 patients.

### Genetic Diagnosis

Genomic DNA was extracted from peripheral blood, collected in EDTA-containing tubes, by standard procedures. Written informed consent for DNA storage and use for genetic analysis and research purposes was obtained from all patients (parents or tutors for patients under age) and relatives, as required by the Ethical Committee of the University of Campania “Luigi Vanvitelli” in accordance with the Declaration of Helsinki. The genetic analysis was performed at the C.S.S.

Mendel Institute of Rome, Italy, through the direct sequencing of *CLCN1* gene and Sanger sequencing. The evaluation of the pathogenic variants was made through the ANNOVAR and Alamut® Software Suite (Interactive Biosoftware). Variants not described in the literature were analyzed by using dedicated softwares, such as Mutation tester, Polyphen2, Provean, M-CAP.

## Results

### Clinical Diagnosis

Clinical data for MC patients are shown in Table 1. Out of 19 patients, 13 are familial with more than one individual affected, while six are isolated cases. Of the latter, one (N17) exhibits a *de novo* mutation not found in her parents. The other five patients (N14-N16, N18-N19) show homozygous or compound heterozygous mutations, whose inheritance was confirmed in their parents.

**Table 1:** Clinical data of MC patients.

**Patient ID**	**Current age in Y**	**Gender**	**Age of onset in years**	**Transient weakness**	**Muscle pain/stiffness**	**Hypertrophy**	**Cold effect**	**Warm Up phenomenon**	**EMG myotonic**	**Clinical course**	**Phenotype**	**Therapy**
N1	69	M	6	Yes	No	Yes	Worse	Yes	Yes	Stable	Thomsen	None
N2	42	F	13	No	No	No	Worse	Yes	Yes	Stable	Thomsen	None\Mex not effective
N3	25	F	6	Yes	No	Yes	Worse	Yes	Yes	Worse	Becker	Mex, effective
N4	28	M	9	Yes	No	Yes	Worse	Yes	Yes	Worse	Becker	Mex, effective
N5	78	F	20	No	Yes	No	No	Yes	Yes	Stable	Thomsen	Mex, effective
N6	36	M	20	No	Yes	No	No	Yes	Yes	Stable	Thomsen	Mex, effective
N7	24	M	4	Yes	No	Yes	Worse	Yes		Stable	Becker	Mex, effective
N8	30	F	5	Yes	No	Yes	Worse	Yes		Stable	Becker	Mex, effective
N9	19	F	asymptomatic								Becker	None
N10	17	M	2	No	No	Yes	Worse	Yes	Yes	Stable	Becker	Mex, effective
N11	70	M	asymptomatic								Thomsen	None
N12	47	M	6	Yes	Yes	Yes	Worse	Yes		Stable	Thomsen	None
N13	27	M	15	Yes	No	Yes	Worse	Yes		Stable	Becker	Mex, effective
N14	53	M	14	Yes	No	Yes	No	Yes		Worse	Becker	Mex, effective
N15	32	M	12	Yes	No	Yes	Worse	Yes		Stable	Becker	Mex, effective
N16	33	F	7	Yes	No	Yes	No	Yes		Worse	Becker	Mex, effective
N17	39	F	10	No	No	Yes	Worse	Yes		Stable	Thomsen	None
N18	17	F	5	No	Yes	Yes	No	Yes		Worse	Becker	None
N19	42	F	4	Yes	Yes	Yes	Worse	Yes		Stable	Becker	None

Twelve patients (63.2%) present Becker type myotonia and seven (36.8%) Thomsen type. Sex ratio M:F in Becker type is 6:6, while in Thomsen myotonia 4:3. The age of onset of the disease ranged from 2 to 15 years in Patients with BM, and from 4 to 20 years in patients with TM. Two patients, one with Thomsen type (N11) and the other with Becker type (N9) are asymptomatic, while their relatives present the typical symptoms of the disease. The warm up phenomenon was present in 100% of patients; transient weakness, referred by 63.1% of all patients, predominates in BM patients (75%) compared with TM patients (26.3%). Muscle pain was present in 42.8% of patients with TM, but in only 16.6% of BM patients. Muscle hypertrophy and cold effect predominates in BM patients (75%) compared to TM patients (50%). The warm up phenomenon is a constant feature in both TM and BM. EMG, available in six patients—three TM and three BM—showed the typical myotonic discharges.

Muscle biopsy—performed on 5/19 patients, two Thomsen and three Becker—showed a normal histological picture in 2, aspecific alterations in 2 and absence of 2B fibers in 1 patient.

### Genetic Analysis

**Figure 1:**
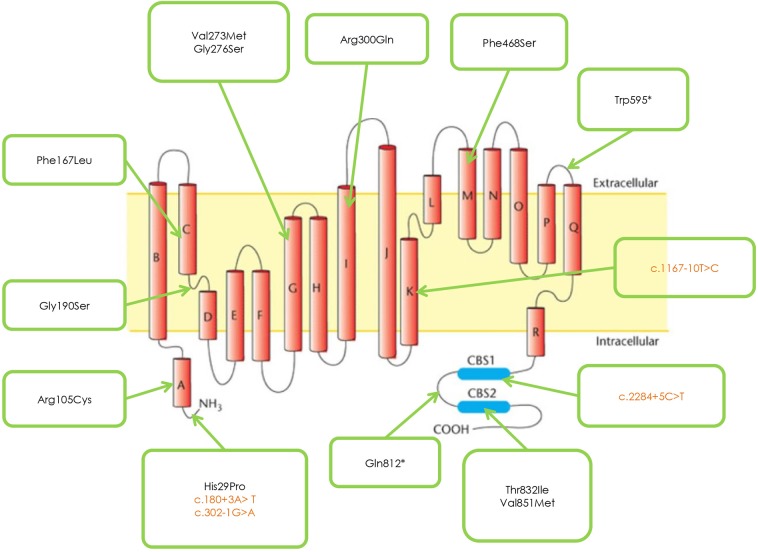
CLCN1 Mutations identified in the studied cohort.

Figure 1 and Table 2 shows the mutations identified in our patients. Out of 18 identified mutations, 14 are located in the coding part of the gene (exons 1, 3, 4, 5, 7, 8, 13, 15, 21, 22) and 4 in the intron part (introns 1, 2, 10, 18). All the exonic mutations but two are missense mutations. Some of them such as p. His29Pro, p.Phe167Leu, p.Val273Met, and p.Val851Met, occur with a relative higher frequency.

**Table 2:** Genetic data of MC patients.

**Patient ID**	**Current age in years**	**Gender**	**Inheritance**	**Relationship**	**Exon/Intron position**	**DNA change**	**Protein**
N1	69	M	AD	Father of N2	ex 1 + ex 22/int 10	c. 86A > C + c.2551G > A in cis/c.1167-10T > C	p. His29Pro + p. Val851Met in cis /?
N2	42	F	AD	Daughter of N1	ex 1 + ex 22/int 10	c. 86A > C + c.2551G > A in cis/c.1167-10T > C	p. His29Pro + p. Val851Met in cis /?
N3	25	F	AR	Niece of N1 and sister of N4	ex 4/int. 10	c.501C > G/c. 1167-10T > C	p.Phe167Leu/?
N4	28	M	AR	Nephew of N1 and brother of N3	ex 4/int. 10	c.501C > G/c. 1167-10T > C	p.Phe167Leu/?
N5	78	F	AD	Mother of N6	ex 7/ex 8 + int 18	c. 817G > A/c.899G > A + c.2284+5C > T in cis	p.Val273Met/p.Arg300Gln + ?
N6	36	M	AD	Son of N5	ex 7	c.817G > A heterozygous	p.Val273Met
N7	24	M	AR	Brother of N8	ex 15	c.1785G > A homozygous	p.Trp595^*^
N8	30	F	AR	Sister of N7	ex 15	c.1785G > A homozygous	p.Trp595^*^
N9	19	F	AR	Sister of N10	int 2/ex 5	c.302-1G > A/c.568-569GG > TC	?/p. Gly190Ser
N10	17	M	AR	Brother of N9	int 2/ex 5	c.302-1G > A/c.568-569GG > TC	?/p. Gly190Ser
N11	70	M	AD	Father of N12	ex 3 + ex 4	c.313C > T + c.501C > G in cis	p.Arg105Cys + p.Phe167Leu in cis
N12	47	M	AD	Son of N11	ex 3 + ex 4	c.313C > T + c.501C > G in cis	p.Arg105Cys + p.Phe167Leu in cis
N13	27	M	AR	2 siblings affected	int 2/ex 7	c.302-1G > A/c.826G > A	?/p.Gly276Ser
N14	53	M	AR		ex 7	c.817G > A homozygous	p.Val273Met
N15	32	M	AR		int 1/ex 21	c.180+3A > T/c.2495C > T	p.THr832Ile
N16	33	F	AR		ex 13	c.1403T > C homozygous	p. Phe468Ser
N17	39	F	AD	*De novo* mutation	ex 22	c.2551G > A heterozygous	p. Val851Met
N18	17	F	AR		ex 1 /ex 7	c.86A > C/c.817G > A	p.His29Pro/p.Val273Met
N19	42	F	AR		ex 21	c.2423C > T homozygous	p.Gln812^*^

* *indicates stop codon point mutation*.

About 70% of mutations are inherited with an AR pattern, the mutation p.Val851Met with an AD pattern, two—p.His29Pro and p.Val273Met – with both mechanisms.

Patients sharing the protein variation p.Gly190Ser have a more severe phenotype than patients with the mutations p. Phe167Leu or Arg105Cys.

Three novel mutations were identified, never described in the literature p. Gly276Ser, Phe486Ser, and p.Gln812^*^, associated with Becker phenotype, two in homozygosis and one in compound heterozygosity. The neuromyological examination of these three patients revealed in the first case (mutation p. Gly276Ser) a slowly progressive Becker phenotype with onset at the age of 16, associated with muscle stiffness, transient weakness cold-aggravated, hand and jaw myotonia; in the second patient (mutation p.Phe486Ser), the onset was earlier, at the age of 7, and the clinical course more severe; in the third patient (mutation p. Gln812^*^), the onset was in childhood, but the disease's course was mild and, at the current age of 42, the patient reports to be stable.

In three unrelated families (N1; N5; N11) two *CLCN1* mutations, inherited in cis on the same allele were identified, in particular *c.86A* > *C* + *c. 2551G* > *A*, (patients N1-N2), *c.313C* > *T* + *501C* > *G* (patients N11-N12), and *c.899G* > *A* + *c.2284*+*5C* > *T* (patient N5).

**Figure 2:**
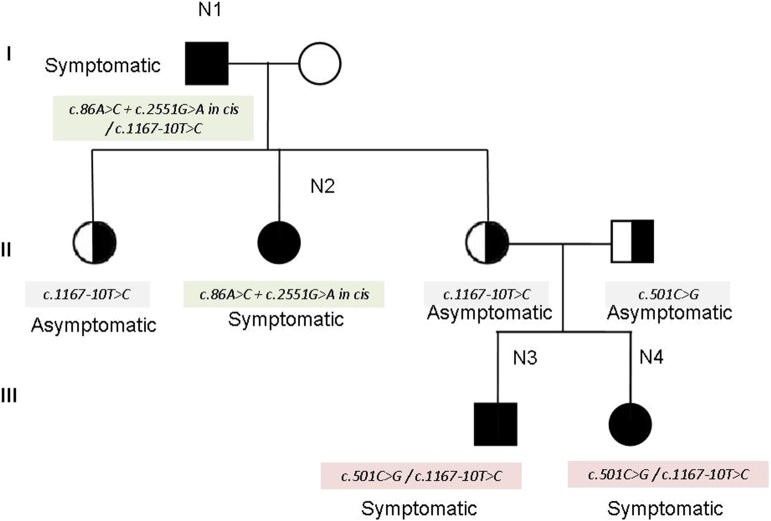
Family tree of individuals N1–N4.

The family N1–N4 (see family tree in Figure 2) deserves particular comment. The father (N1 in the tables) and one of the three daughters (N2 in the tables) show the cis mutation *c.86A* > *C* + *c. 2551G* > *A* (pathogenetic) on one allele, and the mutation c. *1167-10T*> *C* on the other allele. The other two daughters inherited the mutation *c. 1167-10T* > *C* and are clinically unaffected. However, one of the two unaffected daughters had—quite unexpectedly—two children (individuals N3 and N4 in the tables) showing the characteristic symptoms of myotonia since their childhood. The genetic analysis in this part of the family revealed that her husband—unaffected—was a carrier of the mutation *c.501C* > *G* in the CLCN1gene, found in combination with *c. 1167-10T* > *C* in N3 and N4. Therefore, the combination of the mutations *c. 1167-10T* > *C* and *c.501C* > *G* may explain the affected phenotype observed in N3 and N4 (see the family tree). In fact, the mutation *c.501C* > *G* has been already described as affecting or probably affecting function in both compound heterozygosity and in homozygosity (14).

Four mutations found in our cohort of patients, in particular p. Arg105Cys, p.Gly190Ser, p.Thr832Ile, and p.Val851Met were electrophysiologically studied by the group of prof. F. Desaphy and their characteristics published by Altamura et al. (6).

An intra-familial phenotypic variability was observed in two families (patients N9-N10 and N11-N12), one with Thomsen and the other with Becker myotonia. An incomplete penetrance was hypothesized in the Thomsen family, as the father at the age of 70 is still asymptomatic while the son presents symptoms since the age of 6.

Eleven patients were on mexiletine, with an improvement of symptoms; in one patient the drug was ineffective; seven refused therapy. An improvement in muscle symptoms was reported by two patients undergoing steroid therapy for intercurrent diseases.

## Discussion

Mutations in *CLCN1* are widely distributed along the entire gene sequence (12). Unlike the report by Brugnoni et al. (14), who described in a cohort of 106 Italian patients a higher frequency of mutations in exons 4 and 5 of the *CLCN1* gene, mutations in these exons were only seen in 3 of our families.

The mutation p. Gly190Ser, found in both Thomsen and Becker phenotypes, causes a more severe clinical presentation in the latter, compared with mutations p. Phe167Leu or p. Arg105Cys, as demonstrated through the electrophysiological studies by Desaphy et al. (18) and Egushi et al. (14).

The neuromyological examination of the three patients carrying the novel mutations p.Gly276Ser, p.Phe468Ser, and p. Gln812^*^ revealed a different clinical presentation, with a more severe course of the disease associated to the mutation p. Phe486Ser. We report the first case of documented concomitant occurrence of Thomsen and Becker clinical pictures in the same family.

In conclusion, our data, describe a set of *CLCN1* mutations in the population of Southern Italy different from those previously indicated (19–21) in the Italian cohort of MC patients. Furthermore, they report three novel mutations that widen the spectrum of mutations characterizing the clinical picture of these patients.

## Data Availability Statement

The raw data supporting the conclusions of this article will be made available by the authors, without undue reservation, to any qualified researcher.

## Ethics Statement

The studies involving human participants were reviewed and approved by Ethical Committee of University of Campania. Written informed consent for DNA storage and use for genetic analysis and research purposes was obtained from all patients (parents or tutors for patients under age) and relatives, as required by the Ethical Committee of the University of Campania Luigi Vanvitelli, in accordance with the Declaration of Helsinki. Written informed consent for participation was not required for this study in accordance with the national legislation and the institutional requirements.

## Author Contributions

LPo conceived and designed the study and drafted the manuscript with input from the co-authors. RP, PD'A, CO, LPa, and MS performed the clinical diagnosis and follow-up of patients. ME and EP prepared DNA samples for the analysis. AD performed the molecular analysis of the patients, at the CSS Mendel Institute in Rome. All authors approved the final version of the manuscript.

### Conflict of Interest

The authors declare that the research was conducted in the absence of any commercial or financial relationships that could be construed as a potential conflict of interest.
